# Exemplar-Based Image Inpainting Using a Modified Priority Definition

**DOI:** 10.1371/journal.pone.0141199

**Published:** 2015-10-22

**Authors:** Liang-Jian Deng, Ting-Zhu Huang, Xi-Le Zhao

**Affiliations:** School of Mathematical Sciences/Research Center for Image and Vision Computing, University of Electronic Science and Technology of China, Chengdu, Sichuan, P. R. China; Universitat de Valencia, SPAIN

## Abstract

Exemplar-based algorithms are a popular technique for image inpainting. They mainly have two important phases: deciding the filling-in order and selecting good exemplars. Traditional exemplar-based algorithms are to search suitable patches from source regions to fill in the missing parts, but they have to face a problem: improper selection of exemplars. To improve the problem, we introduce an independent strategy through investigating the process of patches propagation in this paper. We first define a new separated priority definition to propagate geometry and then synthesize image textures, aiming to well recover image geometry and textures. In addition, an automatic algorithm is designed to estimate steps for the new separated priority definition. Comparing with some competitive approaches, the new priority definition can recover image geometry and textures well.

## Introduction

**Fig 1:**
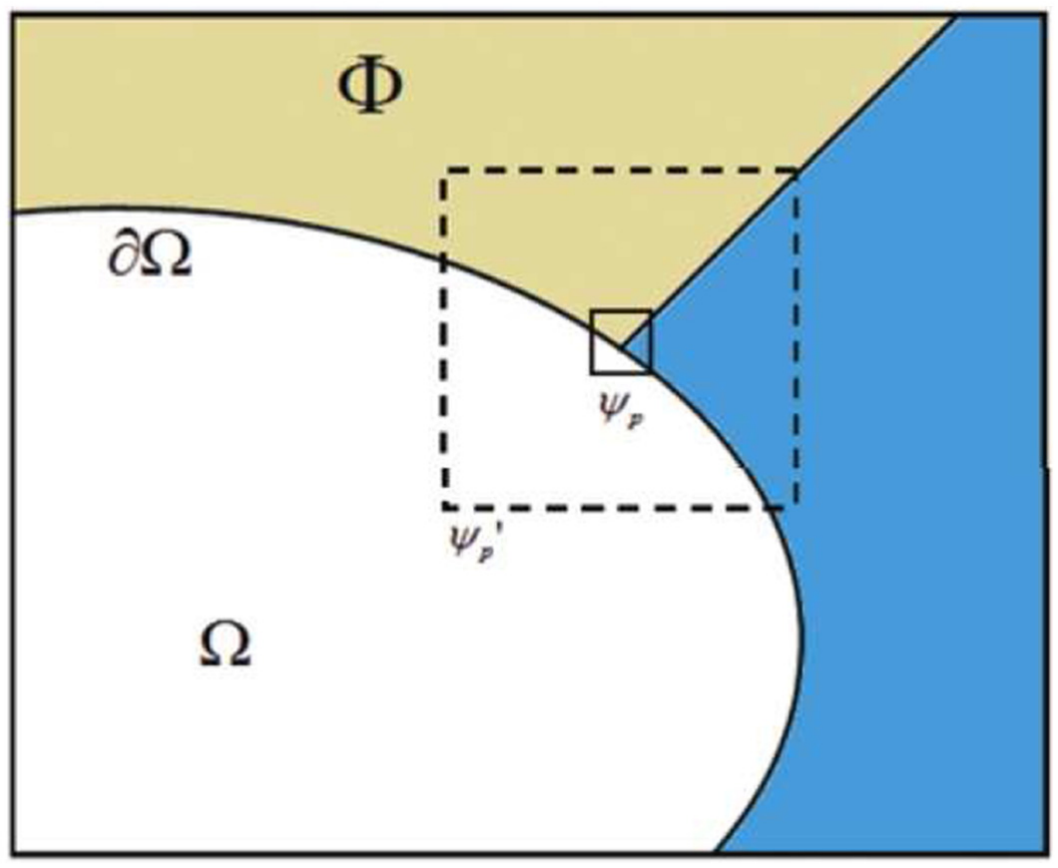
Φ is the source region/image, Ω is the target region and ∂Ω represents the boundary between the source region and the target region. *ψ*_*p*_ is the patch that will be filled, $\psi_{p}^{\prime }$ stands for a bigger patch having the same center *p* with *ψ*_*p*_.

Image inpainting aims to recover the scratches in photograph, repair the damaged regions of an image, remove the specify objects. Users first assign the undesired areas called inpainting domain/target region (see Fig 1), and then make use of an inpainting approach to fill in the corresponding target region of an image that is generally consisted of geometry and textures.

Several approaches have been proposed for image inpainting problems recently. These methods are mainly divided into two categories: partial differential equation (PDE) based approaches and exemplar-based techniques. PDE-based approaches are to construct the diffusion PDE according to the isophote propagation (i.e., propagating the edge information into the target region along the line with same gray values). This technique is first introduced by Bertalmio et al. [1]. It establishes a diffusion PDE so that the boundary information propagates into the target region along the isophote direction. Based on the work of Bertalmio et al., Chan and Shen propose two PDE-based models [2, 3], Total Variation (TV) model and Curvature Driven Diffusion (CDD) model, to deal with the non-texture image inpainting problems. Although these inpainting methods perform well to the images with pure structures, they have to face a same drawback that the diffusion process will lead to some blur, especially when the target region is large.

Exemplar-based techniques [4–12] are a very efficient inpainting method for large target regions. They tend to fill in the target regions by directly copying and pasting patches from source regions, thus image textures are preserved well. Recently, more exemplar-based methods [13–19] for image/video applications are proposed. In [16], Barnes et al. present a novel image editing algorithm PatchMatch to find approximate nearest-neighbor patches from image patches. The motivation of the algorithm is based on that some good patch matches can be found via random sampling, in the meanwhile, the good patch matches can be propagated to surrounding areas. In particular, this interactive technique has been used by Adobe photoshop. In [14], Korman et al. propose coherency sensitive hashing (CSH) that extends locality sensitivity hashing and PatchMatch introduced in [16] to find matching patches between two images. CSH can get quite fast speed and obtain accurate results. He et al. in [15] utilize propagation-assisted KD-trees to compute nearest-neighbor fields. The proposed algorithm can get faster speed than CSH and PatchMatch method. In [17], Newson et al. produce an automatic video inpainting algorithm that extends the PatchMatch method in [16] to the spatio-temporal case. This algorithm is fast and can deal with complex scenes for the video. Wexler et al. in [18] propose a framework to complete the missing information in the video. The completion is viewed as a global optimization problem that is solved by a new proposed algorithm. In [19], Liu et al. utilize multiscale graph cuts algorithm for exemplar-based image inpainting. To reduce the computation, authors present a global energy optimization model for the exemplar-based inpainting, and then solve the model on the low-resolution scale using a proposed multiscale graph cuts algorithm.

For exemplar-based methods, there are very few works to explain the performances from a mathematical point of view, but limited references, e.g., [20–22]. In particular, in [20], Ballester et al. present a novel filling-in algorithm for image inpainting from the point of variational approach. The method employs the joint interpolation of image gray-levels and isophotes directions, to fill in the missing data along the isophot lines. The interpolation is realized by solving a variational problem via its gradient descent flow. In [21], authors explain the theoretical points of exemplar-based methods’ ability to recover well the texture. In addition, Aujol et al. in [22] give the theoretical explanation of exemplar-based image inpainting to the recovery of geometry. In their work, authors propose well-posed variational models that associated to exemplar-based algorithms, and discuss the relations between several optimization models and the original algorithms.

The real-world images are generally consisted of geometry and textures. Criminisi et al. [23, 24] design an exemplar-based inpainting algorithm that combines the advantages of texture synthesis algorithms (e.g., [25–28]) and isophote-based inpainting technique [1]. The priority definition for deciding the filling-in order is important to fill in the missing region. Xu et al. [12] propose an exemplar-based inpainting algorithm based on patch sparse representation. The concept of sparse representation is introduced under the consideration that the missing patches could be represented by the sparse linear combinations of candidate patches. In the meanwhile, they establish a constrained optimization model for solving the image inpainting problems. The work of Hesabi et al. [29] improves the sparse patch propagation based on the contribution of Xu et al. In addition, some methods based on sparse representation such as [30, 31] also have been proposed for image inpainting.

### Related work

Criminisi et al. in their work [23] propose a novel exemplar-based inpainting algorithm. The priority of their algorithm is defined by a confidence term and a data term. If the patch in the target region is with the highest priority, it will be filled in first by searching the most similar patch from the source region. After filling in one patch, the corresponding priority of the filled patch will be updated promptly. In particular, the process is repeated until that the target region is filled completely. The algorithm is detailedly described as follows.

1. For each point *p* on the boundary *δ*Ω (see Fig 1), we set a square patch *ψ*_*p*_ with the center *p*. The patch size is defined flexibly by user according to the practical conditions. In particular, we empirically set the patch size with 9 × 9 pixels in our work.
2. Computing the priority *P*(*p*) for each patch via the following formula,
  $$\begin{array}{c} P(p)=C(p)D(p), \end{array}$$ (1)
  where *C*(*p*) is called confidence term, *D*(*p*) is the data term. They are defined as follows
  $$\begin{array}{c} C\left(p\right)=\frac{\sum_{q\in \psi_{p}⋂\bar{\Omega }}C\left(q\right)}{|\psi_{p}|},D\left(p\right)=\frac{|\nabla_{p}^{⊥}.n_{p}|}{\alpha }, \end{array}$$
  where $\bar{\Omega }$ stands for the complementary set of target region Ω, ∣*ψ*_*p*_∣ is the area of patch *ψ*_*p*_ (i.e., patch area is equal to the number of nonzero elements of the patch), *n*_*p*_ is an unit vector orthogonal to boundary *δ*Ω at the point *p*, $\nabla_{p}^{⊥}$ is an isophote vector and *α* is normalization parameter (*α* = 255 for a gray-level image, see details in [23]). Data term *D*(*p*) plays a role to propagate geometry into the target region, and confidence term *C*(*p*) describes the dependence of *ψ*_*p*_ with its surrounding pixels in the source region. If there are more pixels of source image surrounding the pixel *p*, *C*(*p*) will acquire higher value. In particular, the initialization is that *C*(*p*) = 0, ∀*p* ∈ Ω, $C(p)=1,\forall p\in \bar{\Omega }$ and $D(p)=-0.1,\forall p\in \Omega \cup \bar{\Omega }$ which Ω is the target region.
3. Selecting a patch *ψ*_*p*_ with the highest priority, and filling in the patch by searching the most similar patch $\psi_{\hat{q}}$ from source image Φ. The following equation is used to measure the similarity between two patches,
  $$\begin{array}{c} \psi_{\hat{q}}=\text{arg}\min_{\psi_{q}\in \Phi }d\left(\psi_{p},\psi_{q}\right), \end{array}$$ (2)
  where *d*(*ψ*_*p*_, *ψ*_*q*_) is defined as the sum of squared differences (SSD) of the already filled pixels between the two patches
4. Each pixel *p*′, *p*′ ∈ *ψ*_*p*_ ∩ Ω, is filled by the corresponding pixel in $\psi_{\hat{q}}$.
5. Updating the confidence value with the following formula:
  $$\begin{array}{c} C\left(q\right)=C\left(p\right),\forall q\in \psi_{p}\cap \Omega , \end{array}$$ (3)
6. Repeating phase 1 to phase 5.

We iteratively execute the algorithm until the target region Ω is filled completely. In particular, we define one iteration of the algorithm (i.e., from phase 1 to phase 5) as one “step” that will be frequently used in the following sections.

### Contributions

There are mainly two contributions in this paper.

- *New priority definition to encourage geometry propagation*. Different with Criminisi’s method, we separate Criminisi’s priority definition into two phases, one only formed by the data term *D*(*p*) and the other only formed by the confidence term *C*(*p*). This strategy can prevent image geometry from being destroyed effectively, and reconstruct image textures well. In addition, the proposed priority definition also works well for the case of curved or cross-shaped structures.
- *An automatic algorithm to estimate steps of the new priority definition*. The automatic algorithm is designed according to one important assumption (see details from Eq (5)), and it can determine the steps of the new priority definition fast without using any extra information.

### Overview

The organization of this paper is as follows. First, we give the proposed new separated priority definition for exemplar-based image inpainting. In addition, we also present the corresponding algorithm to automatically estimate steps for the new priority definition. Second, we present extensively visual and computational comparisons with some state-of-the-art exemplar-based inpainting methods. Furthermore, we also discuss the performance under some special cases, e.g., the case when the order of new priority is changed. Finally, we draw the conclusions.

## A new priority for exemplar-based inpainting

### A new priority definition and its motivation

**Fig 2:**
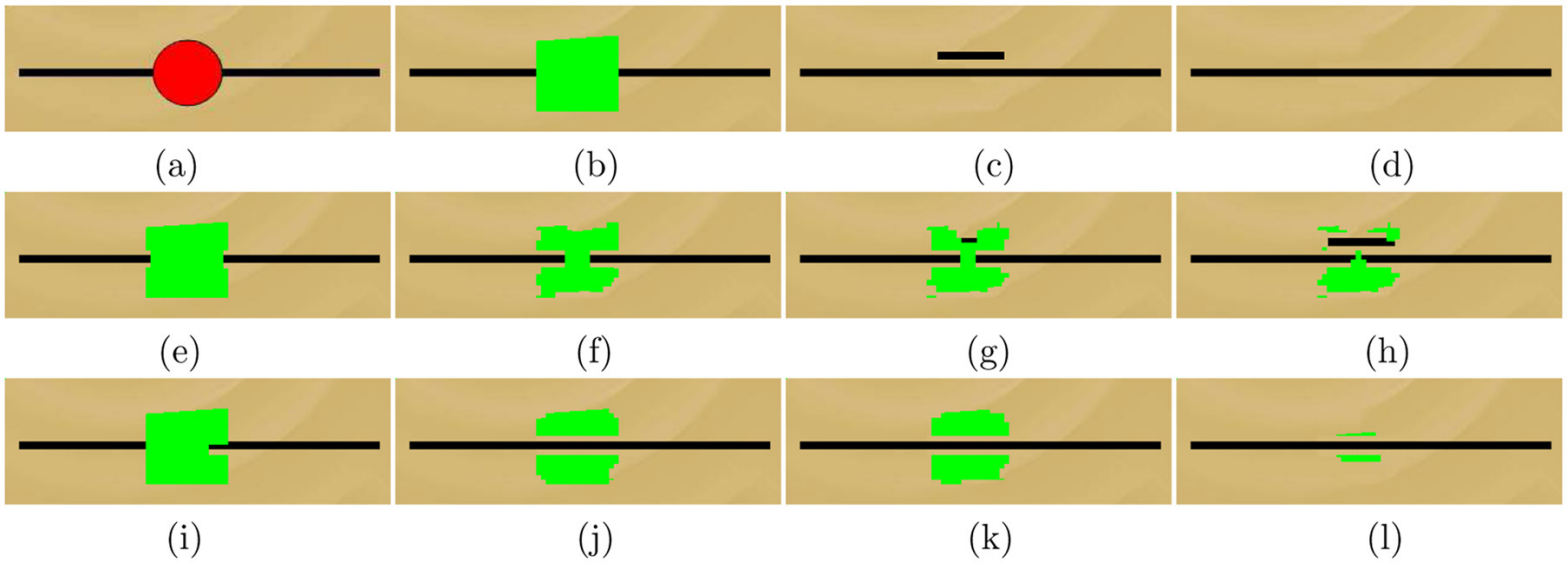
(a) The original image; (b) The green target region; (c) and (d) are the inpainted images by the method in [23] and our method, respectively. (e)-(h) are the process of Criminisi’s method when 5th step, 50th step, 72th step and 100th step, respectively. (i)-(l) describe the process of the proposed method when 5th step, 50th step, 72th step and 270th step, respectively.

An image is generally consisted of geometry and textures. For Criminisi’s method, it tends to propagate the geometry and textures into the target region simultaneously, since the priority definition of Criminisi’s method is determined by two terms, one is the confidence term that encourages textures propagating, and the other is the data term that prefers to propagate geometry. Although the way to propagate geometry and textures simultaneously obtains excellent results, it sometimes appears significant miscopies or makes image geometry being destroyed. For instance, Fig 2(a) is a test image created by author. It is formed by two parts: a black line on the bottom level and a red ball on the top level. From Fig 2(c), Criminisi’s method generates the wrongly short black line from the neighboring long black line due to the improper priority definition. In this paper, we try to design a separated priority definition that is determined by the data term first and then by the confidence term. The new priority can propagate image geometry into the target region first, then synthesize textures. The proposed new definition is given as follows,

$$\begin{array}{c} P\left(p\right)=\{\begin{array}{cc} D(p), & first\;phase,\\ C(p), & second\;phase, \end{array} \end{array}$$ (4)

In particular, we still set the same initializations with Criminisi’s method [23] (see Eq (1)).

### Estimate steps automatically for the new priority definition

For the separated priority definition in Eq (4), how to define the step number for each phase is a quite challenge problem. In our work, we give an estimation algorithm to adaptively determine how many steps carry on for the first phase and the second phase, respectively. Actually, we only need to estimate the steps for the first phase, then executes the second phase until the target region is filled completely. For instance, if the estimated steps are 25 for the first phase, we only need to run the first 25 steps by *P*(*p*) = *D*(*p*), then execute the rest procedure by *P*(*p*) = *C*(*p*). Note that although users also can determine the step number by hand, it is not a convincing choice obviously. In particular, the estimation algorithm is based on a key observation that will be introduced as follows.

#### Assumption based on an observation

**Fig 3:**
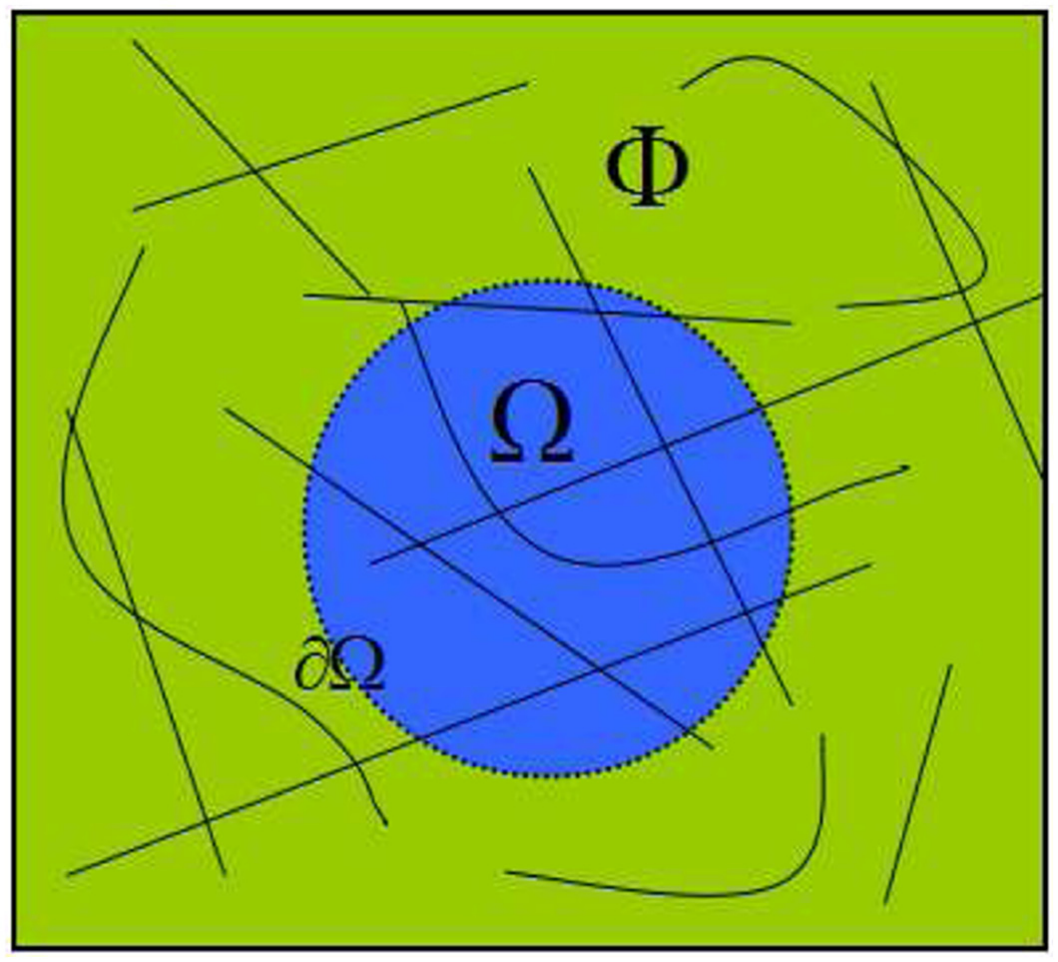
An inpainting image *I* with structure lines (i.e., black solid lines).

**Fig 4:**
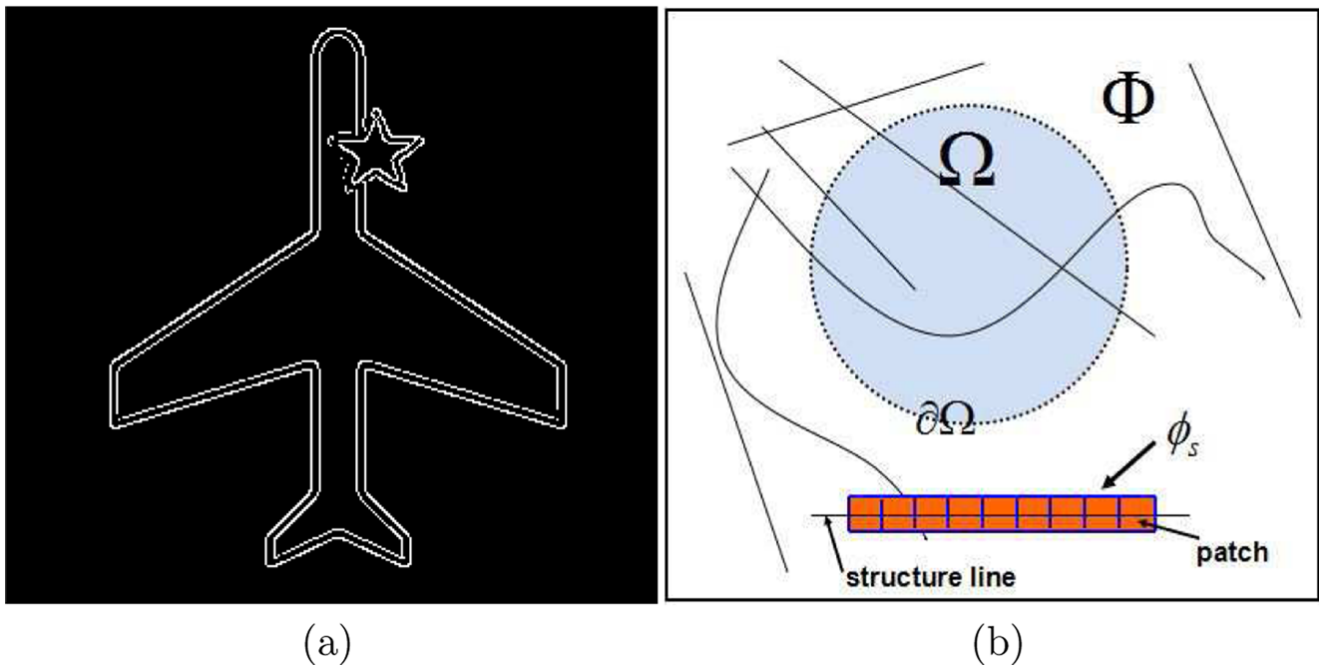
(a) The edge image of “plane” by “canny” edge detector; (b) a structure bar *ϕ*_*s*_ (orange part), blue frames are the filling patches which have a central black structure line (note that we regard edges as structure lines in our work). In particular, *A*_Φ_*s*__ is computed by the sum of all structure bars in Φ (color images are better visualized in the pdf file).

Since we consider that an image *I* is consisted of geometry *I*_*s*_ and textures *I*_*t*_, we have the relation *I* = {*I*_*s*_ ∪ *I*_*t*_∣*I*_*s*_ ∩ *I*_*t*_ = *ϕ*}. From Figs 3 and 4(b), we can see that *I*_*s*_ is consisted of the orange part (i.e., structure bar) and a black solid line. Note that the black solid line is easy to compute via some edge detectors, e.g., “canny” detector or “prewitt” detector. In our work, we select the classic and fast “canny” detector (see details on http://en.wikipedia.org/wiki/Canny_edge_detector) as it is enough for our experiments. Similarly, we define the source region as Φ = {Φ_*s*_ ∪ Φ_*t*_∣Φ_*s*_ ∩ Φ_*t*_ = *ϕ*} and the target region as Ω = {Ω_*s*_ ∪ Ω_*t*_∣Ω_*s*_ ∩ Ω_*t*_ = *ϕ*}, where Φ_*s*_, Ω_*s*_ represent the geometry in Φ and Ω, respectively, and Φ_*t*_, Ω_*t*_ stand for the textures in Φ and Ω, respectively.

Here, we give an assumption for the proposed estimation algorithm, and the assumption is base on a key observation presented as follows:

**Assumption** *For an image, it contains three parts: source region* Φ, *target region* Ω *and the boundary* ∂Ω, *we make the following assumption*:

$$\begin{array}{c} \frac{A_{\Phi_{s}}}{A_{\Phi }}=\frac{A_{\Omega_{s}}}{A_{\Omega }}, \end{array}$$ (5)

where A_*_ represents the area of region *. In particular, we measure the area of one region using the quantity of pixels.

Actually, the target region Ω is generally unknown, e.g., an image with ink, thus we can utilize the rate of the known source region Φ (i.e., $\frac{A_{\Phi_{s}}}{A_{\Phi }}$) to approximately get the rate of the unknown target region Ω (i.e., $\frac{A_{\Omega_{s}}}{A_{\Omega }}$).

#### Details for steps estimation

**Fig 5:**
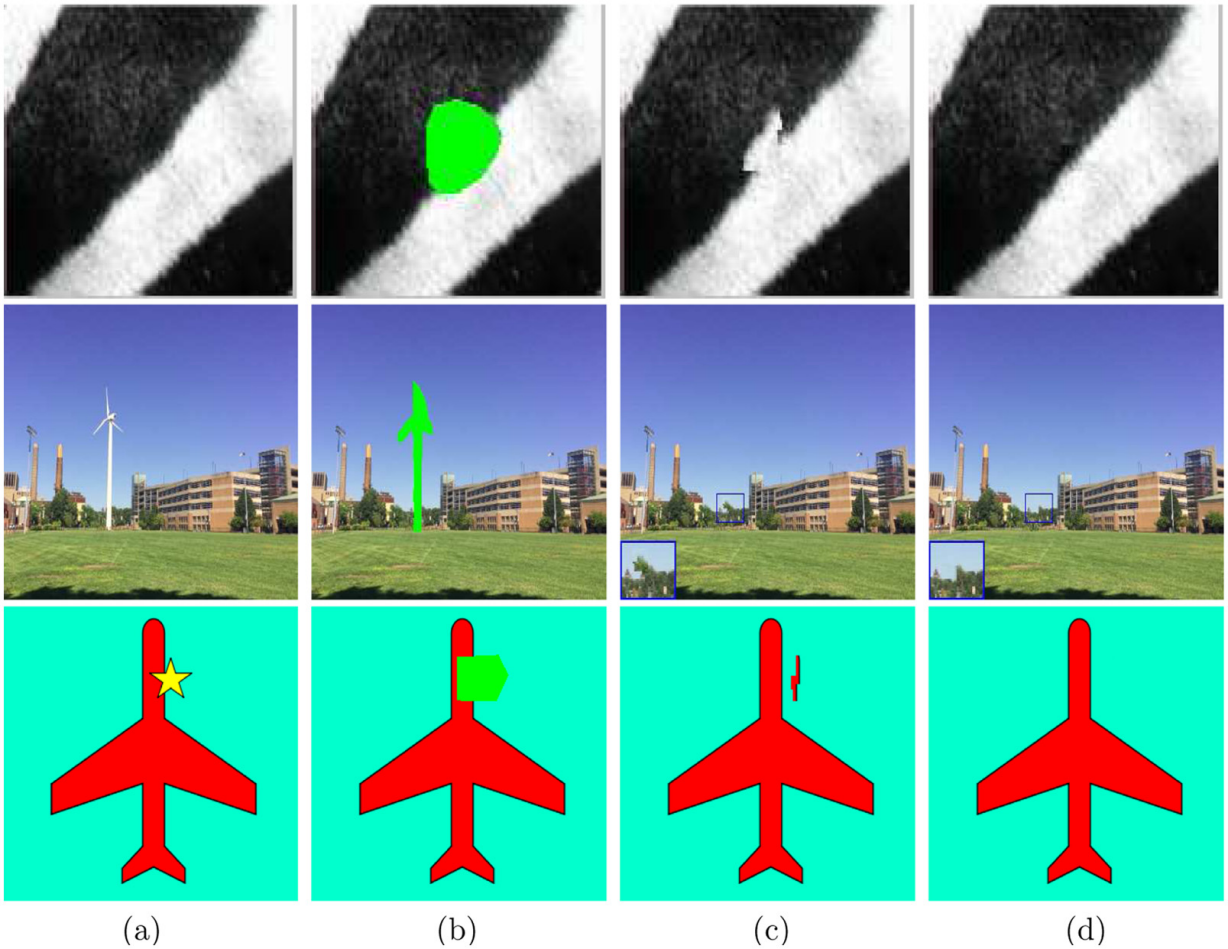
Test images from top to bottom: “zebra” (with the size of 115 × 138), “windmill” (311 × 380) and “plane” (277 × 302). (a) The original images; (b) The original images with the green target regions; (c) and (d) are the inpainted images by the method in [23] and our method, respectively. The first phase of the proposed method executes 16, 45 and 10 steps, respectively. Readers are recommended to zoom in all images for better vision.

From above introduction, we know that geometry *I*_*s*_ is consisted of the orange part (called structure bar here) and a black solid line. We can utilize “canny” detector to compute the black solid line. For instance, Fig 4(a) is an edge map of image “plane” by “canny” method (find “plane” in Fig 5(a)). We can regard the edge map as the black line. In addition, since we consider that the geometry has a width, just like the orange part in Fig 4(b), thus we employ a parameter *ρ* as the width of geometry. We have the following relation,

$$\begin{array}{c} A_{\Phi_{s}}=\rho A_{E_{\Phi }}, \end{array}$$ (6)

where *E*_Φ_ is the edge map of Φ, *A*_*E*_Φ__ is computed by the quantity of nonzero elements in *E*_Φ_. In our work, *ρ* is set to be 9, it is same with the patch size 9 × 9. Furthermore, we also have the relation *A*_Ω_*s*__ = *ρA*_*E*_Ω__, thus Eq (5) is equivalent to the following relation

$$\begin{array}{c} \frac{A_{E_{\Phi }}}{A_{\Phi }}=\frac{A_{E_{\Omega }}}{A_{\Omega }}, \end{array}$$ (7)

**Fig 6:**
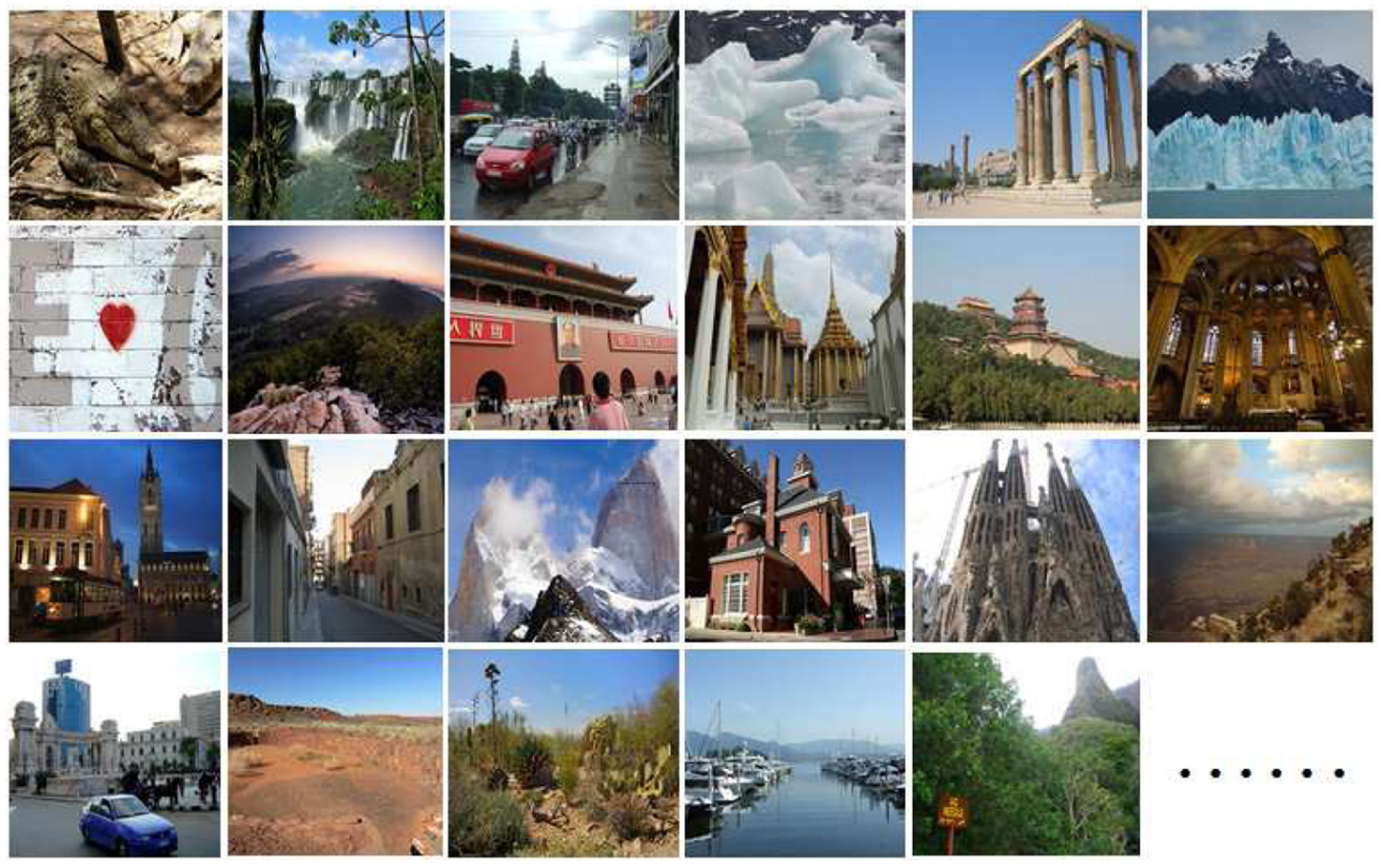
An open dataset (237 images) to test the assumption in Eq (7).

**Fig 7:**
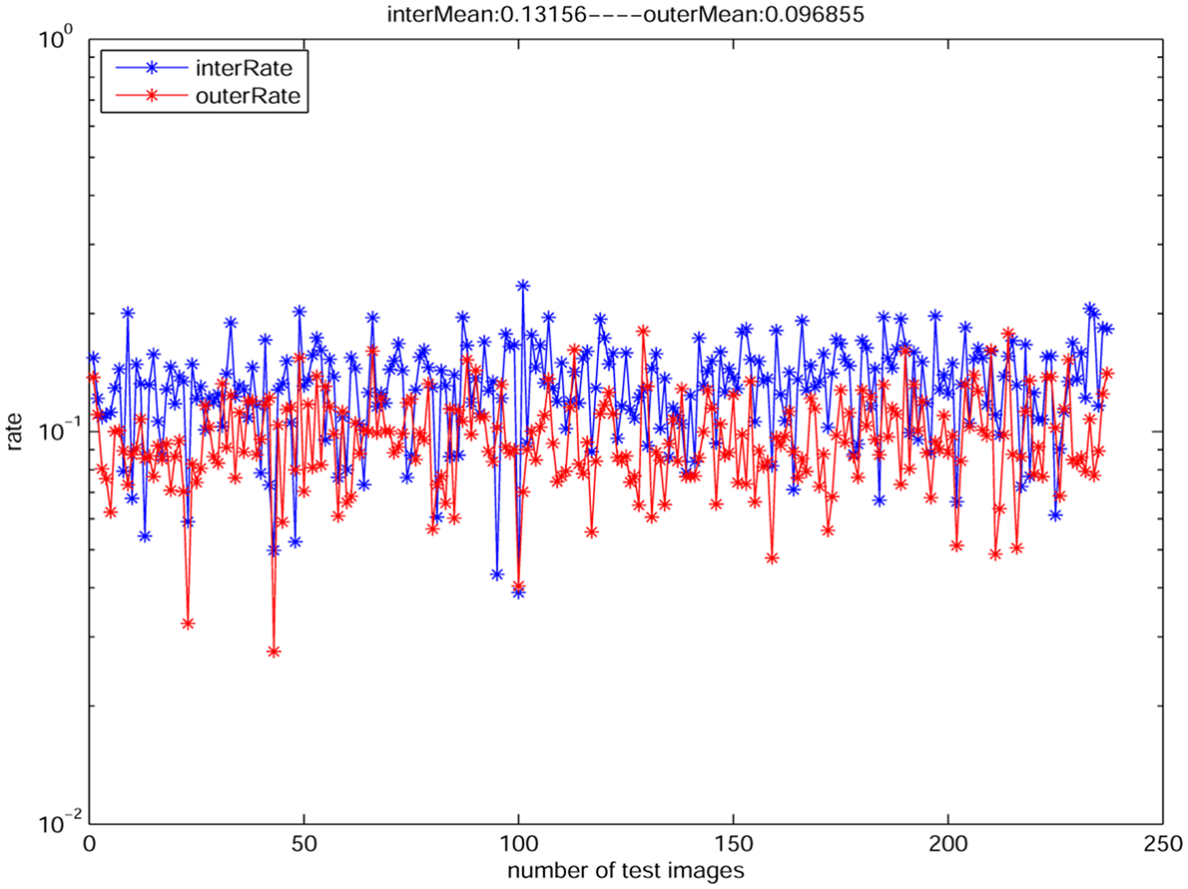
The rates of $\frac{A_{E_{\Omega }}}{A_{\Omega }}$ (blue points) and $\frac{A_{E_{\Phi }}}{A_{\Phi }}$ (red points) for Eq (7) on the open dataset. Note that Eq (7) is reasonable since the red points approach to the blue points. The average rate of the 237 images is 0.132 for the blue points and 0.097 for the red points. The mask is randomly with $\frac{4}{25}$ unknown region.

**Fig 8:**
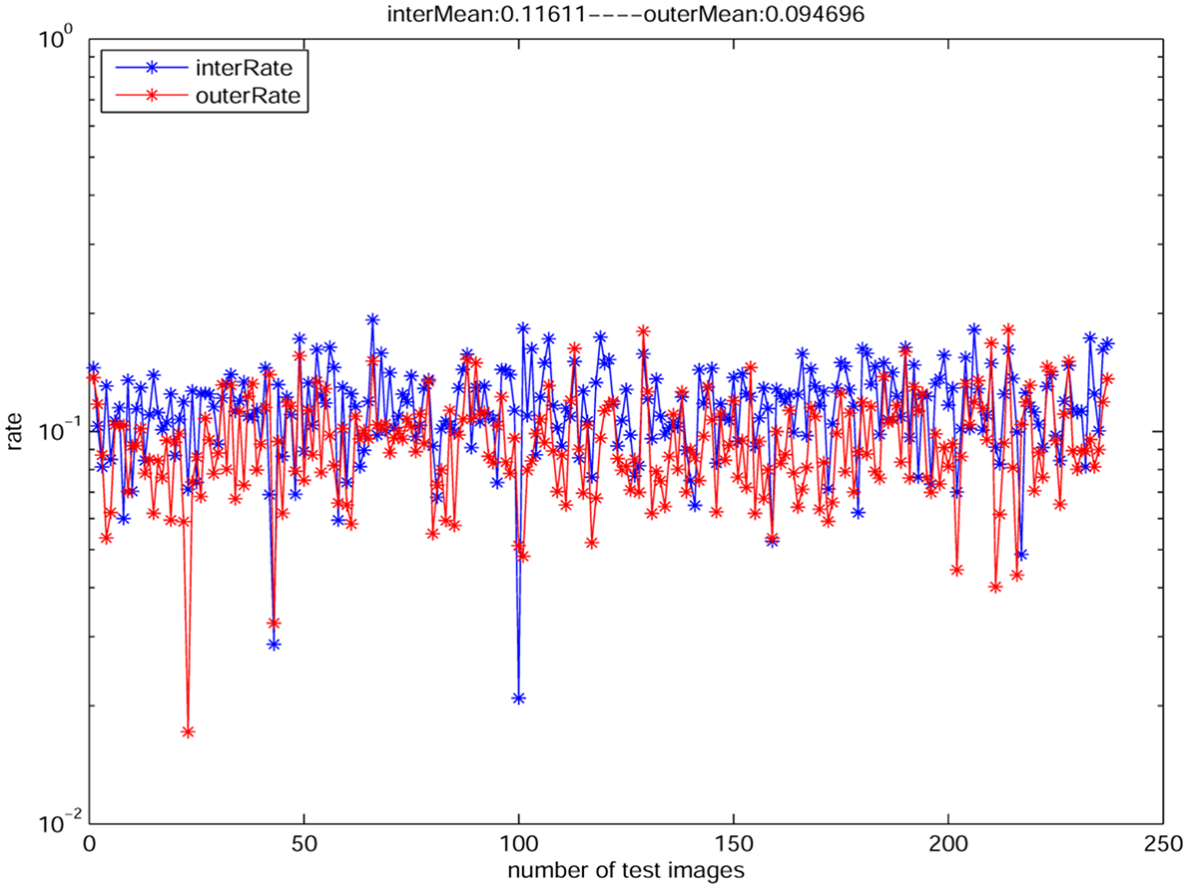
The rates of $\frac{A_{E_{\Omega }}}{A_{\Omega }}$ (blue points) and $\frac{A_{E_{\Phi }}}{A_{\Phi }}$ (red points) with the mask of randomly $\frac{9}{25}$ unknown region. The average rate of the 237 images is 0.116 for the blue points and 0.095 for the red points.

To present the rationality of our assumption, we make a statistic analysis based on an open dataset “im2gps” that contains 237 natural images (see Fig 6, the open dataset “im2gps” is available on http://graphics.cs.cmu.edu/projects/im2gps/). We first compute the edge maps using “canny” detector on the gray channel of the 237 images. Then we compute the rates of $\frac{A_{E_{\Phi }}}{A_{\Phi }}$ and $\frac{A_{E_{\Omega }}}{A_{\Omega }}$, respectively. From Fig 7, we learn that the rate of $\frac{A_{E_{\Phi }}}{A_{\Phi }}$ (red points) is closely to the rate of $\frac{A_{E_{\Omega }}}{A_{\Omega }}$. Thus it proves the rationality of the assumption Eq (7). In particular, in the test, we only select a mask with $\frac{4}{25}$ unknown region for all images, since it is very time-consuming if we create masks for each images. Fig 8 shows the similar result with Fig 7 based on a mask with $\frac{9}{25}$ unknown region. Note that there are some outliers that the two rates are quite different, but actually the number of the outliers is very small, thus we think this case will not influence our assumption very much. Actually, if the mask is with bigger unknown region, the difference between $\frac{A_{E_{\Phi }}}{A_{\Phi }}$ and $\frac{A_{E_{\Omega }}}{A_{\Omega }}$ is smaller.

To estimate steps automatically, we assume the following relationship,

$$\begin{array}{c} \frac{A_{\Omega_{s}}}{A_{\Omega }}=\frac{T_{\Omega_{s}}}{T_{\Omega }}, \end{array}$$ (8)

where *T*_Ω_*s*__ represents the total step number to fill in Ω_*s*_, i.e., the step number of first phase in Eq (4). In addition, the total step number *T*_Ω_ can be estimated by the areas of patch *ψ*_*p*_ and target region Ω, i.e., *T*_Ω_ = *A*_Ω_/(0.5*A*_*ψ*_*p*__). In particular, the parameter 0.5 means that the proposed algorithm averagely propagates a half of patch into the target region Ω for each step. The final step estimation is obtained by Eqs (5), (6) and (8):

$$\begin{array}{c} T_{\Omega_{s}}=\frac{A_{\Phi_{s}}}{A_{\Phi }}T_{\Omega }=2\rho \cdot \frac{A_{E_{\Phi }}A_{\Omega }}{A_{\Phi }A_{\psi_{p}}}, \end{array}$$ (9)

where *A*_Ω_ and *A*_Φ_ are easy to estimate accurately by the known inpainting mask. *A*_*E*_Φ__ and *A*_*ψ*_*p*__ are computed by “canny” edge detector and the known patch size, respectively. *T*_Ω_*s*__ is the finally desired step number for the first phase of the proposed new definition. For the second phase, we only need to execute the exemplar algorithm using *P*(*p*) = *C*(*p*) until that the target region is filled completely. Note that the estimated steps sometimes are not the most accurate value due to the inaccurate edge map, but it can already get good enough results.

Actually, our method is based on the related exemplar-based inpainting in [24]. Although the methods [13–18] also belong to the category of exemplar-based methods, they realize the patch propagation in a quite different way comparing with the method [24] and our method. They realize the patch propagation via PatchMatch, CSH, kd-trees etc., while the method [24] and our method realize patch propagation via defining the priority definition and simple patch searching and copying.

#### Computation reduction using a patch-in-patch strategy

Criminisi’s method [23] gets excellent results for image inpainting, but this approach has to encounter a drawback that it needs expensive computation. Because Criminisi’s method has to search the most similar patch by Eq (2) within the whole source image Φ. In this work, we utilize a simple patch-in-patch approach to reduce the expensive computation. This approach selects the most similar patch within a bigger patch $\psi_{p}^{\prime }$ but the whole source image Φ.

We only need to change Eq (2) slightly to get the new exemplar selection method that is used to measure the similarity between two patches,

$$\begin{array}{c} \psi_{\hat{q}}=\text{arg}\min_{\psi_{q}\in \psi_{p}^{\prime }}d\left(\psi_{p},\psi_{q}\right), \end{array}$$ (10)

where *d*(*ψ*_*p*_, *ψ*_*q*_) is defined as the sum of squared differences (SSD) of the already filled pixels between the two patches *ψ*_*p*_, *ψ*_*q*_, and $\psi_{p}^{\prime }$ is the bigger patch with same center *p* with *ψ*_*p*_ (see Fig 1). We set the patch $\psi_{p}^{\prime }$ with the size of (2*w* + 1) × (2*w* + 1), *w* ∈ N, and the value of *w* will be given in the experiments. We combine the new priority definition with the patch-in-patch strategy to get the final proposed method.

## Results and Discussions

In the section, we employ some images with different masks to test the proposed method. The experimental computer is a laptop with 3.25GB RAM and Intel(R) Core(TM) i3-2370M CPU: @2.40 GHz. We compare the proposed method with some state-of-the-art methods, e.g., Criminisi’s method “04’TIP” [23] (codes available on https://github.com/ikuwow/inpainting_criminisi2004), “07’TPAMI” by Wexler et al. [18] (software available on http://www.wisdom.weizmann.ac.il/˜vision/VideoCompletion.html), Photoshop CS5 [16] and “13’TIP” by Liu et al. [19]. Note that the method “07’TPAMI” is a part of the *Content Aware Fill* feature in Photoshop CS5 which has been optimized. In addition, we also present the computation comparisons for the different methods. In the experiments, we empirically set *w* = 70 for examples “ball”, “ErieLake” and *w* = 30 for the rest of examples. Actually, tuning *w* slightly for each examples can get better performance, but for simplicity, we mainly set two choices of *w*, i.e., *w* = 30 and *w* = 70. For the fairness, we set same *w* in the experiments both for Criminisi’s method and the proposed method. All test images in the experiments are created by author’s PowerPoint or taken by author’s camera and cellphone, and these images (denoted as “TestImages”) are available on http://www.escience.cn/people/dengliangjian/Data.html. Furthermore, matlab codes for the proposed method are available on http://www.escience.cn/people/dengliangjian/codes.html.

### Results

From Fig 2, the target region (see the green region in Fig 2(b)) is not a regular circle but an arbitrary region covering a red ball. The inpainted image by Criminisi’s method “04’TIP” [23] (Fig 2(c)) causes the mismatch of short black line while the proposed method removes the red ball completely. The second row and third row show the inpainting process of Criminisi’s method [23] and the proposed method, respectively. In particular, 14 steps, estimated automatically by the proposed estimation algorithm, are taken for the first phase. From Fig 2(e)–2(h), since the propagation of geometry and textures are implemented simultaneously, it is easy to cause the mismatch from the surrounding areas. For instance, the short black line is wrongly copied from the long black line. On the contrary, the proposed method propagates the geometry into the target region only for the first 14 steps (see Fig 2(i) and 2(j)), then synthesizes the textures (see Fig 2(k) and 2(l)), thus the red ball is removed completely. This experiment demonstrates that the proposed new priority definition can preserve image geometry well.

From Fig 5, we compare the proposed method with Criminisi’s method “04’TIP” [23]. From the figure, the proposed method performs better, because it protects the image geometry well. For instance, in the first row, the geometry between black textures and white textures is preserved well and the proposed method fills the green hole completely (see (d) in the first row). Similarly, the image “plane” in the third row also preserves the straight line structure via the proposed method, while Criminisi’s method causes obvious miscopies (see (c), (d) in the third row). In the second row of Fig 5, the proposed method can remove the windmill completely, Criminisi’s method however slightly copies wrong patches from the source region (see the close-ups). Note that the proposed algorithm automatically estimates 16, 45 and 10 steps for the first phase of the new priority definition.

**Fig 9:**
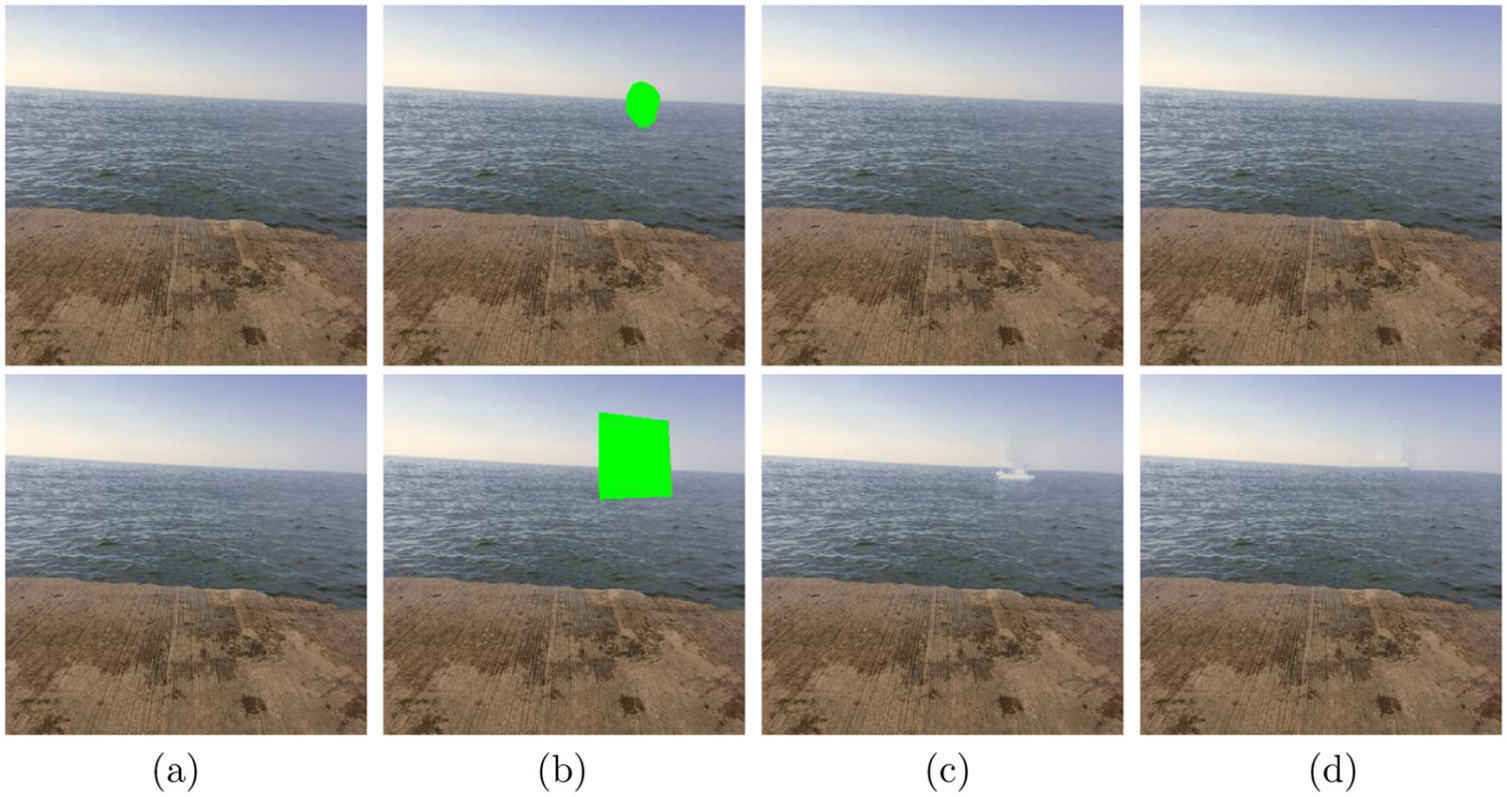
Test images from top to bottom: “ErieLake” (321 × 455) and “ErieLake2” (321 × 455). (a) The original images; (b) The original images with different green target regions; (c) and (d) are the inpainted images by the method in [23] and our method, respectively. The results demonstrate that the proposed method is robust to various target regions.

In Fig 9, we compare our method with Criminisi’s method “04’TIP” using different target regions. From the first row, we learn that the two method both performs well if the target region is small. However, in the second row, the proposed method recovers the image well when giving a larger target region, while Criminisi’s method leaves significant miscopies. It demonstrates that the proposed method is more robust to varying and large target regions than Criminisi’s method.

**Fig 10:**
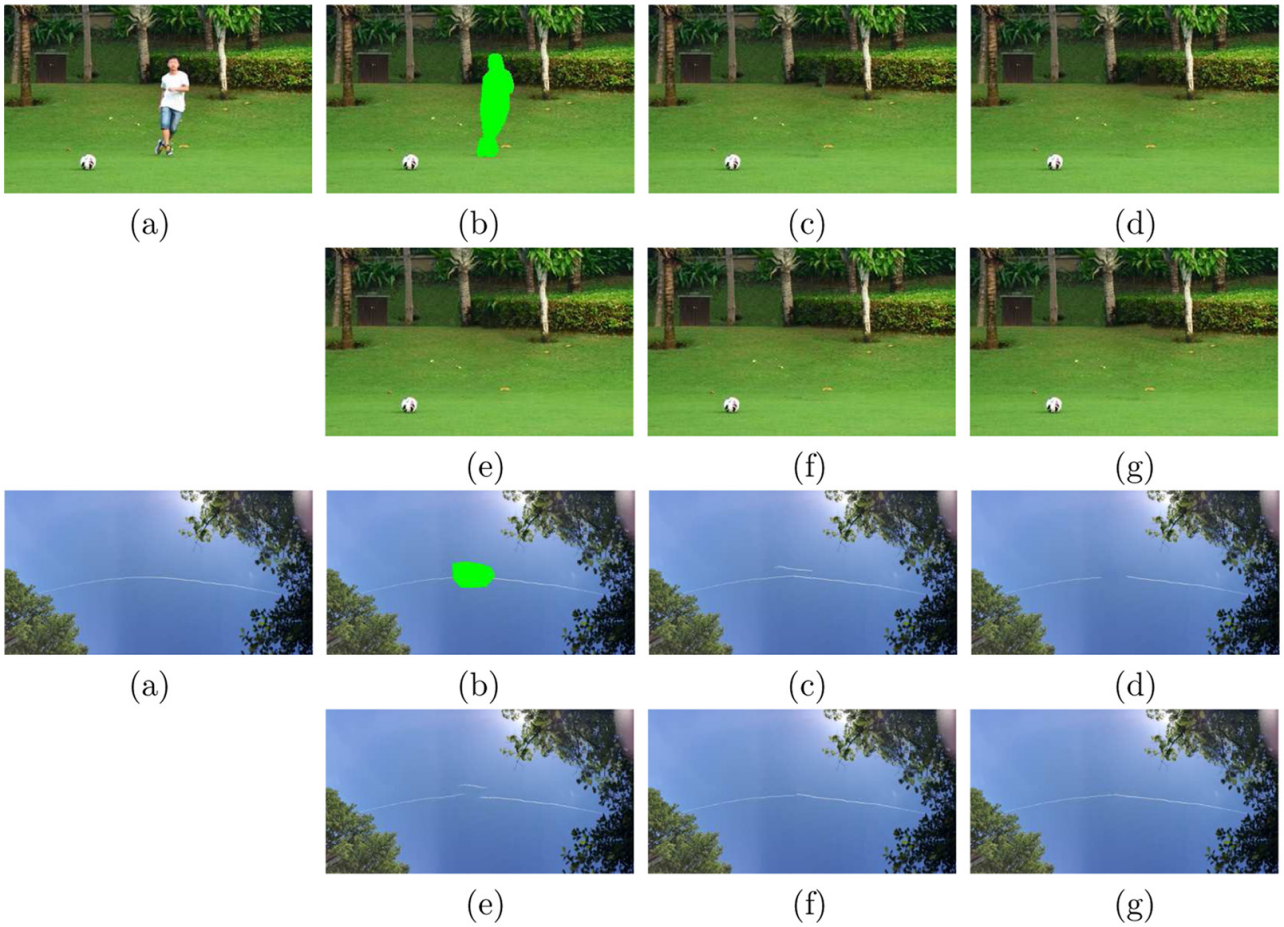
Test images from top to bottom: “kicking” (213 × 343) and “sky” (210 × 407). (a) The original images; (b) The original images with the red target regions; (c) Criminisi’s method “04’TIP” [23]; (d) The method “07’TPAMI” by Wexler et al. [18]; (e) Photoshop CS5 [16]; (f) The method “13’TIP” by Liu et al. [19]; (g) The proposed method. Note that the man in the first example is one of authors, he consents that the image is freely available. The individual in this manuscript has given written informed consent (as outlined in PLOS consent form) to publish these case details.

In Fig 10, we employ two natural images taken by author’s camera and cellphone, to test the performance of different methods. From the figure, we know that all methods perform well for the first example but Criminisi’s method “04’TIP”, since it causes miscopies on the target region. For the second example, we need to recover the white smoke line from the green target region (here *w* = 40 can get better performance). However, the methods “04’TIP” and Photoshop CS5 create wrong copies of white smoke and “07’TPAMI” breaks the white smoke line obviously. The method “13’TIP” recovers the white smoke line well, but still slightly worse than the proposed method.

We also present the computation comparisons for the different methods. Since the proposed method is similar to Criminisi’s method “04’TIP”, we first show the computation comparisons of the two methods. Note that we only apply the patch-in-patch strategy to the proposed method rather than “04’TIP”, aiming to show the computation reduction of patch-in-patch strategy. In Table 1, we present some computation comparisons for different test images. Due to the expensive computation of searching optimal exemplars (i.e., “GE” in Table 1), we employ MEX file to accelerate the phase “GE”. For the fairness, Criminisi’s method and our method all use MEX file in this phase. From Table 1, we learn that the proposed method costs significantly less computation than Criminisi’s method. We also test the computation time when giving different target regions, e.g., “ErieLake” in Fig 9. It is easy to know that the computation time depends on the size of target region, if the target region is small, the corresponding computation time will be less.

**Table 1:** Computation time of Criminisi’s method [23] and our patch-in-patch method. For simplicity, “Ready phase”(RP), “Compute gradients” (CG), “Get priorities” (GP), “Get exemplars” (GE), “Copy&Update” (CU). In particular, the phase “RP” of the proposed method includes the procedure of estimating steps. “ErieLake2” and “Curveline2” represent the computation time in the second row of Fig 9 and the second row of Fig 11, respectively. (Time unit: second.)

Image	Method	RP	CG	GP	GE	CU	Total
Plane	Criminisi’s	0.26	1.12	0.63	28.82	0.13	30.96
	Proposed	1.26	1.99	1.19	1.06	0.22	**5.72**
ErieLake	Criminisi’s	0.38	1.24	0.22	26.04	0.12	28.00
	Proposed	1.09	1.46	0.33	3.50	0.13	**6.51**
ErieLake2	Criminisi’s	0.15	4.27	1.39	31.99	0.41	38.21
	Proposed	0.51	4.00	1.77	2.99	0.39	**9.66**
Windmill	Criminisi’s	0.19	1.80	0.78	42.62	0.18	45.57
	Proposed	1.79	2.36	1.25	1.04	0.26	**6.70**
Curveline	Criminisi’s	0.17	0.18	0.35	4.9	0.02	5.62
	Proposed	0.87	0.22	0.42	0.62	0.03	**2.16**
Curveline2	Criminisi’s	0.19	0.24	0.65	7.20	0.03	8.31
	Proposed	0.71	0.29	0.78	0.73	0.04	**2.55**
Circle	Criminisi’s	0.59	6.58	0.75	91.87	0.39	100.18
	Proposed	1.26	9.47	1.06	0.87	0.63	**13.29**

In addition, we also compare our method with other state-of-the-art methods in Table 2. Since we can not measure the time of Photoshop CS5 accurately, thus we do not compare it in Table 2. However, when we execute image inpainting on Photoshop CS5, we note that Photoshop CS5 is significantly the fastest method since it has been optimized. From Table 2, the proposed method obtains the smallest computation except the first example “circle”. The method “13’TIP” gets the smallest computation for the first example. In particular, the computation also depends on the size of target region. For instance, the bigger target region gets more computation (see the second and third rows of Table 2).

**Table 2:** Computation time of different methods: Criminisi’s [23], “07’TPAMI” [18], Photoshop CS5 [16], “13’TIP” [19] and the proposed method. Note that we can not measure the time of Photoshop CS5 accurately, but it is the fastest method. (Time unit: second.)

Image	Criminisi’s	07’TPAMI	13’TIP	Proposed	Photoshop CS5
Circle	100.18	402.50	9.40	13.29	-
Curveline	5.62	19.54	16.01	2.16	-
Curveline2	8.31	26.01	21.54	2.55	-
Kicking	18.74	171.54	7.01	4.85	-
sky	12.84	70.54	6.81	3.02	-

### Discussions

#### Curved structures propagation

**Fig 11:**
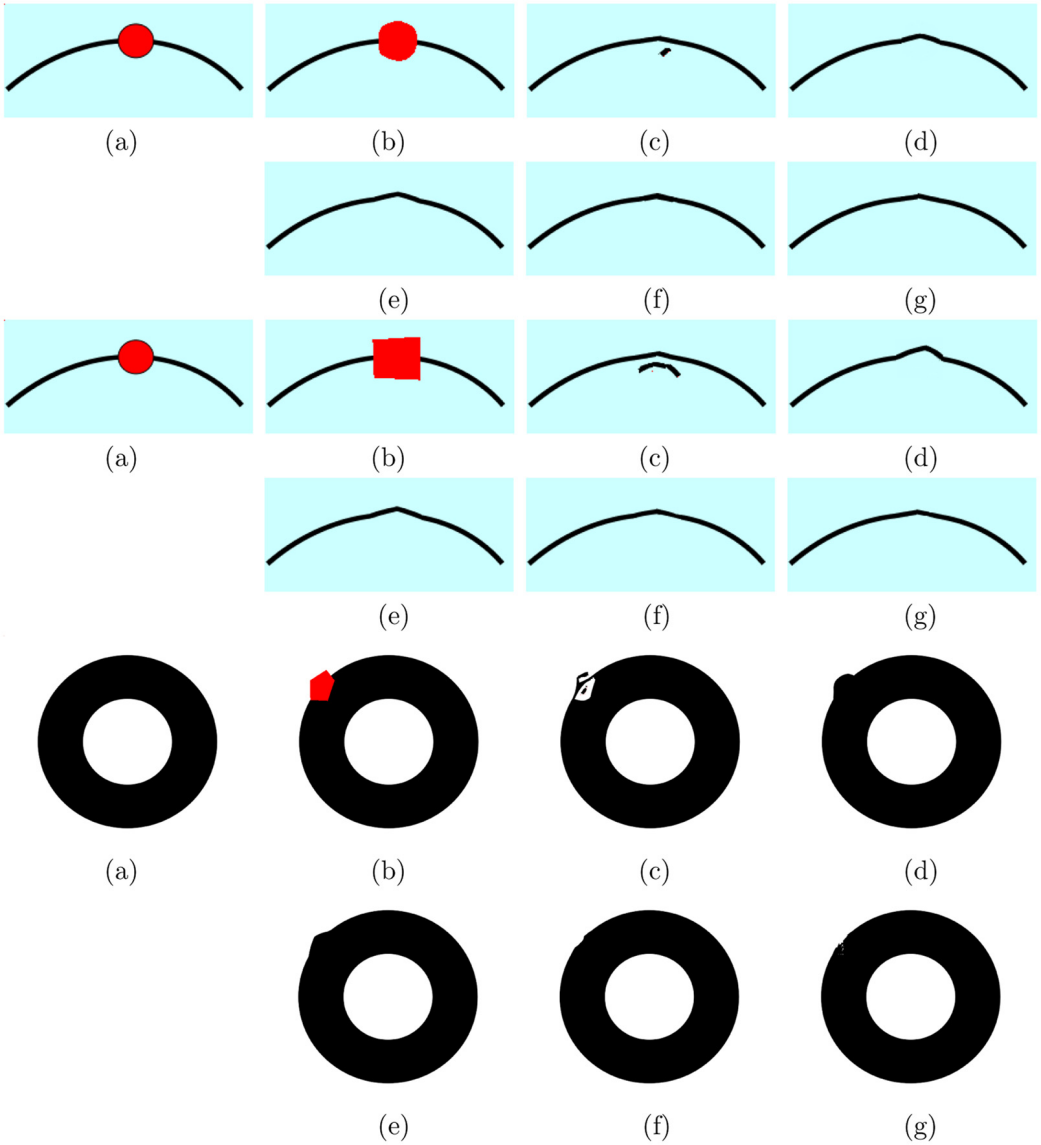
Test images from top to bottom: “curveball” (105 × 233), “curveball2” (105 × 233) and “circle” (454 × 547). (a) The original images; (b) The original images with the red target regions; (c) Criminisi’s method “04’TIP” [23]; (d) The method “07’TPAMI” by Wexler et al. [18]; (e) Photoshop CS5 [16]; (f) The method “13’TIP” by Liu et al. [19]; (g) The proposed method.

In Fig 5, it demonstrates that the proposed method can preserve geometry well, especially for the straight line structures. Here, we tend to discuss the performance of the proposed method for the curve structures. We compare our method with some state-of-the-art exemplar-based inpainting methods, e.g., “07’TPAMI” [18], Photoshop CS5 [16] and “13’TIP” [19]. In Fig 11, we create three images with curve structures, e.g., curve black lines in the first two images and curve edges in the third image. From the first two examples of Fig 11, Criminisi’s method causes significant miscopies and slightly changes the curve black lines. “07’TPAMI”, Photoshop CS5 and “13’TIP” remove the red ball well, but cause a little non-smoothing recovery in the middle of the black line. In particular, the proposed method also removes the red ball completely and gets slightly better performance than the three methods. In the third example of Fig 11, we compare different methods on the case of occlusive edges. The proposed method recovers image edges better than the methods “04’TIP”, “07’TPAMI” [18] and Photoshop CS5 [16], but slightly worse than the method “13’TIP”. Actually, the compared methods all have to face a limitation for the case of curve structures. For instance, they can not recover the curve black line completely for the first two examples of Fig 11, there is a slightly non-smoothing point in the middle of the black line. According to the definition of $D(p)=\frac{|\nabla_{p}^{⊥}.n_{p}|}{\alpha }$, *D*(*p*) in our new priority definition will get larger value at the point *p* that is on the straight line, since the angle between $\nabla_{p}^{⊥}$ and *n*_*p*_ is small so that we can get larger $|\nabla_{p}^{⊥}.n_{p}|$. Thus the given algorithm prefers to propagate patches along the straight line, in the meanwhile, it also obtains relatively good inpainted results comparing to other methods. We can conclude that the proposed method performs better for straight linear structures but curved structures.

#### The order of priority definition

**Fig 12:**
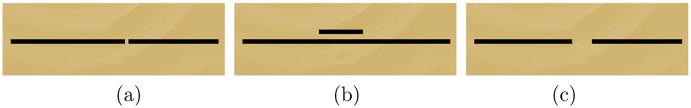
(a) The inpainted image when the first phase uses *C*(*p*) and the second phase uses *D*(*p*) (first phase executes 120 steps); (b) The inpainted image when the first phase uses *C*(*p*)*D*(*p*) and the second phase uses *D*(*p*) (first phase executes 120 steps); (c) The inpainted image when the priority is determined only by confidence term *C*(*p*).

We have proposed the new priority definition and the visual results show the effectiveness of the proposed priority definition. However, if we exchange the order of the first phase and the second phase (i.e., the first phase defined only by confidence term *C*(*p*) and the second phase defined only by data term *D*(*p*)), it can not obtain excellent results (see Fig 12(a)). From Fig 12(b) and 12(c), if the priority of the first phase is defined as *C*(*p*)*D*(*p*) or *C*(*p*), it will appear some miscopied black lines and the geometry will be broken. For the proposed definition, the geometry will be protected properly in the first phase, thus we can only synthesize the textures in the second phase (see Fig 2(d)). Thus the order of the new priority definition in Eq (4) should not be changed.

#### Cross-shaped case and limitations

**Fig 13:**
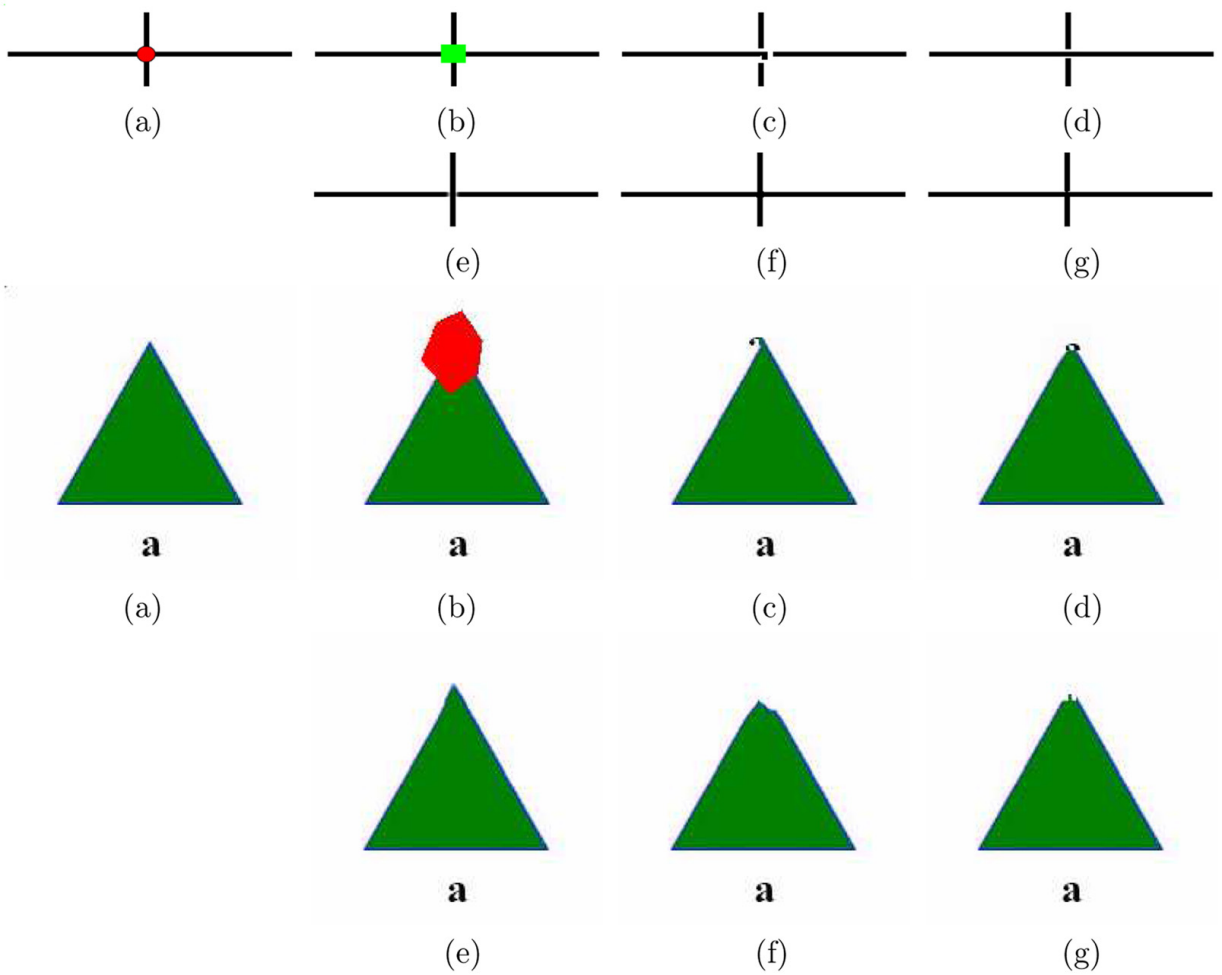
Test images from top to bottom: “cross-shape” (110 × 317) and “triangle” (182 × 184). (a) The original images; (b) The original images with the red target regions; (c) Criminisi’s method “04’TIP” [23]; (d) The method “07’TPAMI” by Wexler et al. [18]; (e) Photoshop CS5 [16]; (f) The method “13’TIP” by Liu et al. [19]; (g) The proposed method.

For the first example of Fig 13, we present the visual results of different methods for the cross-shaped case. From the first example, we know that the method “13’TIP” performs best while other methods including the proposed method break the cross-shaped black line. From the second example of Fig 13, we learn that the proposed work encounters a limitation that it can not recover the edge corner completely. Since the proposed method is sensitive to the geometry that directions change strongly, e.g., the edge corner of the triangle in the second example. In particular, Photoshop CS5 recovers the painted image completely and performs best than other state-of-the-art methods.

## Conclusions

In this paper, we presented a new separated priority definition for exemplar-based image inpainting. The proposed method could handle inpainting problems with large target regions. We also proposed an automatic algorithm to estimate the steps for the separated priority definition. To reduce the computation, we incorporated a common patch-in-patch strategy into the proposed method. Furthermore, we also discussed the computational and visual performance of different exemplar-based methods. The proposed method performed well to recover the geometry but could not recover curved or cross-shaped structures completely. Nevertheless, the proposed method showed better visual results than other compared exemplar-based methods for the case of curved or cross-shaped structures. In particular, our method performed not so well for the case of that geometry changed direction strongly, e.g., the corner of triangle. In addition, the proposed method also obtained competitive computation comparing with other state-of-the-art inpainting methods.
